# Intrusive Mental Images Resembling Psychosis in a Patient With Obsessive-Compulsive Disorder

**DOI:** 10.7759/cureus.111784

**Published:** 2026-06-30

**Authors:** Shaurya P Kushwaha, Maghav Sharma, Shradha Puri

**Affiliations:** 1 General Physician, Vishwas Hospital, Sunam, IND; 2 Psychiatry, OSF HealthCare, Behavioral and Mental Health, Urbana, USA; 3 Psychology, Maharishi Markandeshwar University (MMU), Solan, IND

**Keywords:** intrusive images, mental compulsions, obsessional thoughts, obsessive-compulsive disorder, psychosis differential

## Abstract

Obsessive-compulsive disorder (OCD) may be present with distressing intrusive thoughts, urges, or images. When the content is violent, sexual, religious, or linked to magical thinking, severe OCD can be mistaken for psychosis. We present a 19-year-old man with a six-year history of ego-dystonic intrusive harm-related thoughts and images focused on family members, along with checking, touching, counting, reassurance seeking, religious mental rituals, and sexual obsessions. Symptoms began at age 13 after he read about asthma, and later developed a recurrent fear that his maternal grandmother might develop the illness. He recognized these thoughts and images arising from his own mind, experienced them as distressing and excessive, and used rituals to reduce anxiety. Examination did not elicit hallucinations, externally located perceptions, thought insertion, withdrawal, broadcasting, passivity phenomena, behavioral disorganization, or mood, substance-related, or neurological syndrome. The diagnosis was obsessive-compulsive disorder, International Statistical Classification of Diseases and Related Health Problems10th Revision* *(ICD-10), F42.2 mixed obsessional thoughts and acts. Fluvoxamine 50-150 mg/day was associated with a subjective 10%-20% improvement without documented adverse effects; the treatment was later switched to fluoxetine because of cost-related factors. Long-term follow-up and standardized severity scores were unavailable. This case highlights the importance of assessing thought ownership, resistance, insight, conviction, and the anxiety-reducing function of rituals before labeling vivid intrusive mental images as hallucinations or delusions.

## Introduction

Obsessive-compulsive disorder is characterized by obsessions and compulsions. Obsessions are recurrent and persistent thoughts, urges, or images that are intrusive, unwanted, and associated with distress or anxiety. Compulsions are repetitive behaviors or mental acts that a person feels driven to perform in response to an obsession or according to rigid rules, usually to reduce distress or prevent a feared event [1]. Compulsions may be visible, such as checking or washing, or covert, such as counting, praying, mental reviewing, or neutralizing thoughts [1,2]. Taboo obsessions may involve aggressive, sexual, or religious content and are often associated with shame, concealment, and mental rituals [3,4]. Magical thinking refers to linking neutral actions, numbers, objects, or thoughts with feared outcomes despite little or no realistic causal connection [5,6]. These features can be clinically confusing when the content is vivid, culturally shaped, or religiously charged.

Psychosis broadly refers to impaired reality testing and may include hallucinations, delusions, thought alienation, passivity phenomena, disorganized thought or behavior, and negative symptoms. Psychotic perceptual symptoms are experienced as perceptions without an external stimulus, often with an external location or sensory quality. In contrast, obsessions are usually recognized as self-generated, intrusive, distressing, resisted, and followed by anxiety-reducing rituals [7,8]. The research gap addressed by this case is practical and phenomenological, as vivid intrusive mental images in OCD may resemble psychosis unless clinicians specifically examine ownership, insight, resistance, conviction, and ritual function [7]. Misclassification may expose patients to unnecessary antipsychotic-centered treatment and delay first-line OCD care, including selective serotonin reuptake inhibitors and exposure and response prevention [9-11].

## Case presentation

A 19-year-old unmarried man presented with a six-year illness of insidious onset and continuous course. Symptoms began at age 13 while he was in class 7. He recalled reading about asthma in a book and, two to three weeks later, developed a recurrent thought that his maternal grandmother might develop asthma. No major psychosocial stressors or a clear precipitating life event were elicited apart from this initial cognitive trigger. The fears were focused on close family members and relatives, and a similar preoccupation with his own health was not documented. No serious chronic illness among family members was documented, although an aunt later became acutely unwell, which became incorporated into his fears.

The earliest neutralizing behavior involved touching vehicles. He began touching vehicles whenever the thought recurred because he felt that touching might prevent the feared illness and reduce uneasiness, although he recognized this belief as unreasonable. Within one to two months, the thoughts recurred throughout much of the day during routine activities such as eating, changing clothes, and studying. Soon afterward, he developed religious obsessions and mental rituals. He imagined divine figures blessing and protecting relatives, repeated prayers, and later used spitting rituals to neutralize distress. In early 2002, after an aunt became ill, he repeatedly imagined a protective steel cover around her. He also feared that changing an old idol could reduce divine protection for relatives. These acts were repetitive, ego-dystonic, distressing, and performed to prevent feared harm rather than as ordinary worship.

In late 2002, approximately one year after the onset, he developed recurrent intrusive fears that his maternal uncle might die. To reduce distress, he mentally replaced the relative with another similar-looking person and reassured himself that the dead person was not his uncle. This produced relief for approximately 30 to 60 minutes. At approximately age 16, checking compulsions began. He repeatedly checked locks, and later gas stoves and money, often touching the locks and internally reassuring himself that it was closed correctly. He also developed symmetry and magical-thinking symptoms involving object positions, numbers, and repeated touches. Sexual obsessions were also documented. He reported unwanted sexual thoughts involving a goddess while cleaning or praying before idols, followed by distress, self-disgust, resistance, and compensatory mental imagery of divine forgiveness.

During the last one to two months before the presentation, the frequency of repetitive thoughts, doubts, images, and rituals increased, and these symptoms occupied more time. He reported having only one to two hours for study and daily chores because much of the day was occupied by symptoms. He continued social interactions, and a friend suggested a psychiatric evaluation. Self-care, sleep, and appetite were preserved.

Past psychiatric history was negative. At the initial presentation, he was not documented to be taking psychiatric medication. Past medical history was negative for diabetes mellitus, tuberculosis, asthma, jaundice, and hypertension. No previous major hospitalization, operation, trauma history, or long-term medication use was documented. Family history was negative for psychiatric illness, substance use, suicide, and documented major chronic medical illness. Birth and early childhood developmental history were unavailable. He had good academic and peer relationships, respectful behavior toward teachers, no reported school behavioral complaints, and had been working in a marketing job for one month. Premorbid temperament was not documented.

Mental status examination showed an adequately groomed, conscious, alert, and cooperative young man with good rapport. Speech was coherent and goal-directed but overelaborative, with repeated reassurance seeking. Affect was anxious, reactive, and congruent. Thought assessment revealed harm-related obsessions, sexual obsessions, religious obsessions, symmetry and magical-thinking symptoms, mental rituals, checking, counting, touching, and reassurance seeking. Perception showed no abnormality. Orientation, attention, memory, intelligence, judgment, and higher mental functions were intact. Insight was recorded as 4 out of 5.

**Figure 1:**
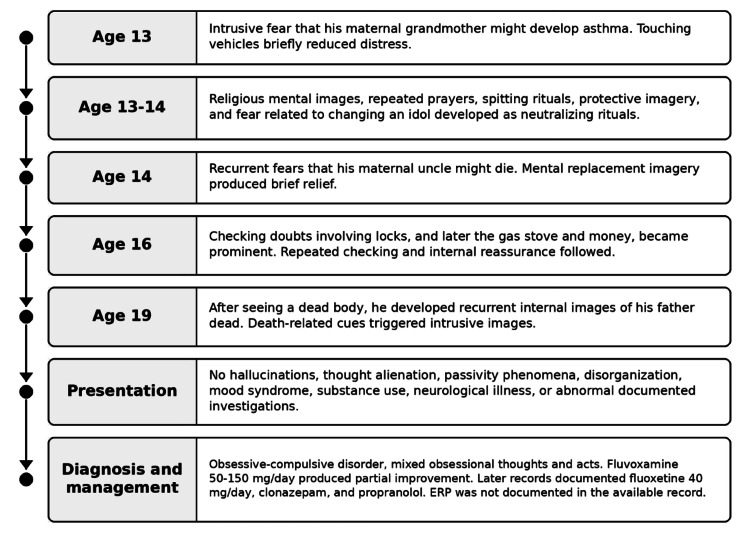
Timeline of symptoms, assessment, investigations, and management Figure 1 was prepared with ChatGPT (OpenAI, 2025, ChatGPT (GPT-5.5), https://chatgpt.com/) AI-assisted formatting support using de-identified case information. The authors reviewed and verified the figure for clinical accuracy.

There was no evidence of hallucinations, externally located perceptions, thought insertion, thought withdrawal, thought broadcasting, passivity phenomena, gross disorganization, catatonia, sustained delusional conviction, depressive syndrome, manic syndrome, substance use, or neurological illness. The clinical examination and investigation record are summarized in Table 1. The timeline is shown in Figure 1.

**Table 1:** Clinical examination and investigations of the patient

Parameter	Finding
Blood pressure	124/74 mm Hg, left arm, sitting position
Pulse	74/min, regular, normal volume
Temperature	Afebrile
General examination	No pallor, icterus, cyanosis, clubbing, pedal edema, lymphadenopathy, thyromegaly, skin lesions, or raised JVP documented
Cardiovascular system	S1 and S2 normal
Respiratory system	Bilateral vesicular breath sounds
Abdominal examination	Soft; liver and spleen not palpable
Central nervous system	Within normal limits
Extrapyramidal symptoms	Absent
Laboratory investigations	Not documented in the available retrospective record
Radiological investigations	Not documented in the available retrospective record
Psychometric assessment	No Y-BOCS or other standardized severity score documented; clinical insight recorded as 4/5

The final clinical diagnosis was obsessive-compulsive disorder, International Statistical Classification of Diseases and Related Health Problems 10th Revision (ICD-10) F42.2, mixed obsessional thoughts and acts. This diagnosis was supported by recurrent intrusive thoughts, urges, and images, along with checking, counting, touching, reassurance seeking, and mental rituals. These symptoms were associated with distress, resistance, partial to good insight, and functional impairment. The differential diagnoses considered and the reasons for excluding them are summarized in Table 2.

**Table 2:** Differential diagnosis of patient symptoms OCD: Obsessive-compulsive disorder.

Differential diagnosis	Why considered	Reasoning
Schizophrenia-spectrum psychosis	Vivid internal images, unusual religious content, evil figures, and magical thinking.	Not supported: no hallucinations, externally located perceptions, thought alienation, passivity, disorganization, or fixed delusional conviction.
Delusional disorder	Neutral actions were linked to feared harm prevention.	Not supported: conviction was not fixed; beliefs were anxiety-driven, partly recognized as unreasonable, resisted, and tied to compulsive neutralization.
Major depressive disorder with intrusive ruminations	Thoughts involved death and harm.	Not supported: no sustained low mood, anhedonia, neurovegetative depressive syndrome, or self-harm ideation.
Bipolar disorder or manic episode	Religious themes and increased mental activity could be misread as mood pathology.	Not supported: no elevated mood, decreased need for sleep, grandiosity, pressured speech, impulsive spending, or manic syndrome.
OCD, ICD-10 F42.2 mixed obsessional thoughts and acts	Obsessions, mental rituals, checking, counting, touching, reassurance seeking, distress, resistance, and insight.	Best overall fit.

The patient was initially started on fluvoxamine 50 mg/day, which was later increased to 150 mg/day. The available retrospective record did not specify the exact dose frequency, titration interval, or number of days after each dose increase. He reported subjective partial improvement of approximately 10% to 20% without documented adverse effects. The record did not specify which symptom domains improved, although later documentation noted a perceived benefit from the medication. The medication was changed because of the cost. The exact number of days from initial prescription to medication change was not documented.

The last available treatment record documented fluoxetine 40 mg/day, propranolol 10 mg three times daily, and clonazepam 1 mg at bedtime. Earlier documentation listed clonazepam 0.5 mg, but the dosing schedule was not specified. No cognitive behavioral therapy (CBT) or exposure and response prevention referral was documented. This should not be interpreted as evidence against exposure and response prevention. Instead, it reflects the limitations of the retrospective record and possible access or resource constraints. Long-term follow-up, adherence, duration of adequate medication exposure, standardized response assessment, and quality-of-life outcomes were unavailable.

## Discussion

The presented case is clinically important because covert rituals and taboo obsessions are not rare in OCD. Mental rituals have been reported in approximately 9.8% to 25% of clinical OCD samples, and some studies report even higher rates among patients without obvious overt compulsions [2]. Unacceptable or taboo thoughts, including sexual, religious, and aggressive obsessions, are also recognized as an OCD symptom dimension and are commonly associated with mental rituals [3,4]. Because these symptoms may involve vivid images, culturally shaped religious content, feared harm, or sexual material, patients may describe them in ways that appear unusual or bizarre. If the clinician focuses only on symptom content rather than insight, ownership, resistance, and the anxiety-reducing function of rituals, severe OCD may be mistaken for psychosis [7].

Hallucinations are perception-like experiences that occur without an external stimulus and usually have sensory force. In psychosis, they are often experienced as externally located. Delusions are fixed false beliefs held with strong conviction despite contrary evidence. Thought insertion, withdrawal, and broadcasting refer to experiences in which thoughts are perceived as being placed into the mind, removed from the mind, or known to others. Passivity phenomena involve feelings, impulses, or actions experienced as controlled by an external force. Disorganized behavior refers to markedly impaired goal-directed behavior. Sustained delusional conviction refers to a fixed belief that is not explained by cultural context or anxiety-driven doubt [7,8]. These features were not documented in this patient.

The treatment distinction is clinically important. OCD treatment prioritizes selective serotonin reuptake inhibitors (SSRIs) or clomipramine and cognitive-behavioral therapy with exposure and response prevention (ERP) [9-11]. ERP is a CBT method in which patients gradually face feared cues while refraining from overt or covert rituals [10]. Psychotic disorders usually require antipsychotic-centered treatment, assessment of safety and functioning, and management of hallucinations, delusions, disorganization, and negative symptoms. Mislabeling OCD as psychosis may therefore delay ERP and lead to unnecessary antipsychotic exposure.

## Conclusions

Vivid intrusive mental images, magical thinking, religious themes, and elaborate mental rituals may resemble psychotic phenomena. In this case, self-generated thought ownership, ego-dystonic distress, resistance, partial to good insight, and temporary relief after neutralization supported obsessive-compulsive disorder, ICD-10 F42.2 mixed obsessional thoughts and acts, rather than a primary psychotic disorder. The absence of hallucinations, externally located perceptions, passivity phenomena, thought alienation, disorganization, and fixed delusional conviction further supported this distinction. Accurate diagnosis matters because management should prioritize SSRIs and CBT with ERP rather than unnecessary antipsychotic-centered treatment. 

## References

[1] Hirschtritt ME, Bloch MH, Mathews CA (2017). Obsessive-compulsive disorder: advances in diagnosis and treatment. JAMA.

[2] Sibrava NJ, Boisseau CL, Mancebo MC, Eisen JL, Rasmussen SA (2011). Prevalence and clinical characteristics of mental rituals in a longitudinal clinical sample of obsessive-compulsive disorder. Depress Anxiety.

[3] Brakoulias V, Starcevic V, Berle D (2013). The characteristics of unacceptable/taboo thoughts in obsessive-compulsive disorder. Compr Psychiatry.

[4] Siev J, Steketee G, Fama JM, Wilhelm S (2011). Cognitive and clinical characteristics of sexual and religious obsessions. J Cogn Psychother.

[5] Siev J, Baer L, Minichiello WE (2011). Obsessive-compulsive disorder with predominantly scrupulous symptoms: clinical and religious characteristics. J Clin Psychol.

[6] Einstein DA, Menzies RG (2004). Role of magical thinking in obsessive-compulsive symptoms in an undergraduate sample. Depress Anxiety.

[7] Oulis P, Konstantakopoulos G, Lykouras L, Michalopoulou PG (2013). Differential diagnosis of obsessive-compulsive symptoms from delusions in schizophrenia: a phenomenological approach. World J Psychiatry.

[8] American Psychiatric Association (2022). Diagnostic and Statistical Manual of Mental Disorders, Fifth Edition, Text Revision (DSM-5-TR).

[9] Janardhan Reddy YC, Sundar AS, Narayanaswamy JC, Math SB (2017). Clinical practice guidelines for obsessive-compulsive Disorder. Indian J Psychiatry.

[10] Hezel DM, Simpson HB (2019). Exposure and response prevention for obsessive-compulsive disorder: a review and new directions. Indian J Psychiatry.

[11] Soomro GM, Altman D, Rajagopal S, Oakley-Browne M (2008). Selective serotonin re-uptake inhibitors (SSRIs) versus placebo for obsessive compulsive disorder (OCD). Cochrane Database Syst Rev.

